# Endovascular treatment of contained ruptured internal thoracic artery aneurysm mimicking a tumor in a patient with neurofibromatosis type 1: a case report

**DOI:** 10.1186/s40792-024-02002-9

**Published:** 2024-08-30

**Authors:** Ryoma Oda, Daisuke Endo, Takeshi Udagawa, Shingo Okada, Ryohei Kuwatsuru, Minoru Tabata

**Affiliations:** 1 Department of Cardiovascular Surgery, Juntendo University Hospital, Tokyo, Japan; 2 Department of Radiology, Juntendo University Hospital, Tokyo, Japan

**Keywords:** Endovascular treatment, Ruptured internal thoracic artery aneurysm, Neurofibromatosis type 1

## Abstract

**Background:**

An internal thoracic artery aneurysm (ITAA) is an exceedingly rare condition, with approximately two-thirds of reported cases being iatrogenic pseudoaneurysms. The remainder are attributed to various causes, including vasculitis, connective tissue disease, and neurofibromatosis type 1 (NF-1). NF-1 is an autosomal dominant disorder characterized by distinct clinical manifestations that occasionally include life-threatening vascular complications. Although NF-1 patients may develop various vascular abnormalities, ruptured ITAA is rarely reported, with only seven published cases.

**Case presentation:**

A 32-year-old man with NF-1 consulted for a three-day history of persistent left back and upper arm pain. Initial chest radiography indicated left pleural effusion and an opacity at the left lung apex. Computed tomography scan revealed a mass in the left upper mediastinum that was initially suspected to be a tumor. Subsequent contrast-enhanced computed tomography revealed the mass to be a subclavian artery aneurysm. Detailed contrast-enhanced computed tomography with 1-mm slices was performed for surgical planning, identifying the mass as a left ITAA with contained rupture. Given the risk of re-rupture, emergency angiography was performed, which confirmed rupture of the left ITAA without extravasation. The ITAA was successfully treated with multiple microcoils at the proximal and distal ends. The patient had an uneventful recovery and was discharged on the fourth postoperative day.

**Conclusions:**

This case highlights the importance of considering vascular lesions in NF-1 patients who present with pleural effusion. It also emphasizes the challenges in diagnosing ITAA and the effectiveness of thin-slice contrast-enhanced computed tomography scans and endovascular treatment.

## Background

An internal thoracic artery aneurysm (ITAA) is a rare disease. Of the 40 cases reported from 1973 to 2012, approximately two-thirds were iatrogenic pseudoaneurysms associated with sternotomy or placement of central venous catheters or pacemaker leads, while the remaining cases were due to vasculitis, connective tissue diseases, neurofibromatosis type 1 (NF-1), fibromuscular dysplasia, and atherosclerosis, or were idiopathic [1]. NF-1 is an autosomal dominant hereditary disorder caused by an abnormality in the long arm of chromosome 17 (17q11.2), occurring in approximately 1 in every 3000 births. Typical clinical manifestations include café-au-lait macules, freckling, peripheral neurofibromas, Lisch nodules, and skeletal abnormalities. Vascular involvement is uncommon, affecting only 3.6% of patients, but can be life-threatening [2]. Although patients with NF-1 exhibit a wide spectrum of vascular abnormalities [3], only seven cases of ruptured ITAA in NF-1 patients have been reported [4–10].

Herein, we present a case of a contained rupture of an ITAA in a patient with NF-1, which posed a diagnostic challenge owing to its similarity to a tumor. Thin-slice contrast-enhanced computed tomography (CE-CT) proved useful for diagnosis, and successful endovascular treatment was achieved with coil embolization.

## Case presentation

**Fig. 1:**
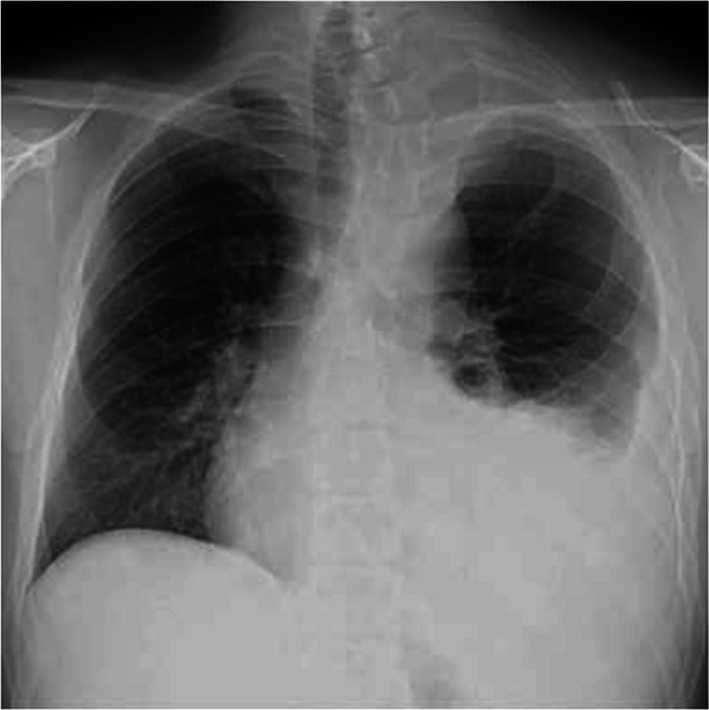
Preoperative chest radiograph. Chest radiography shows a left-sided pleural effusion and an opacity at the apex of the left lung

**Fig. 2:**
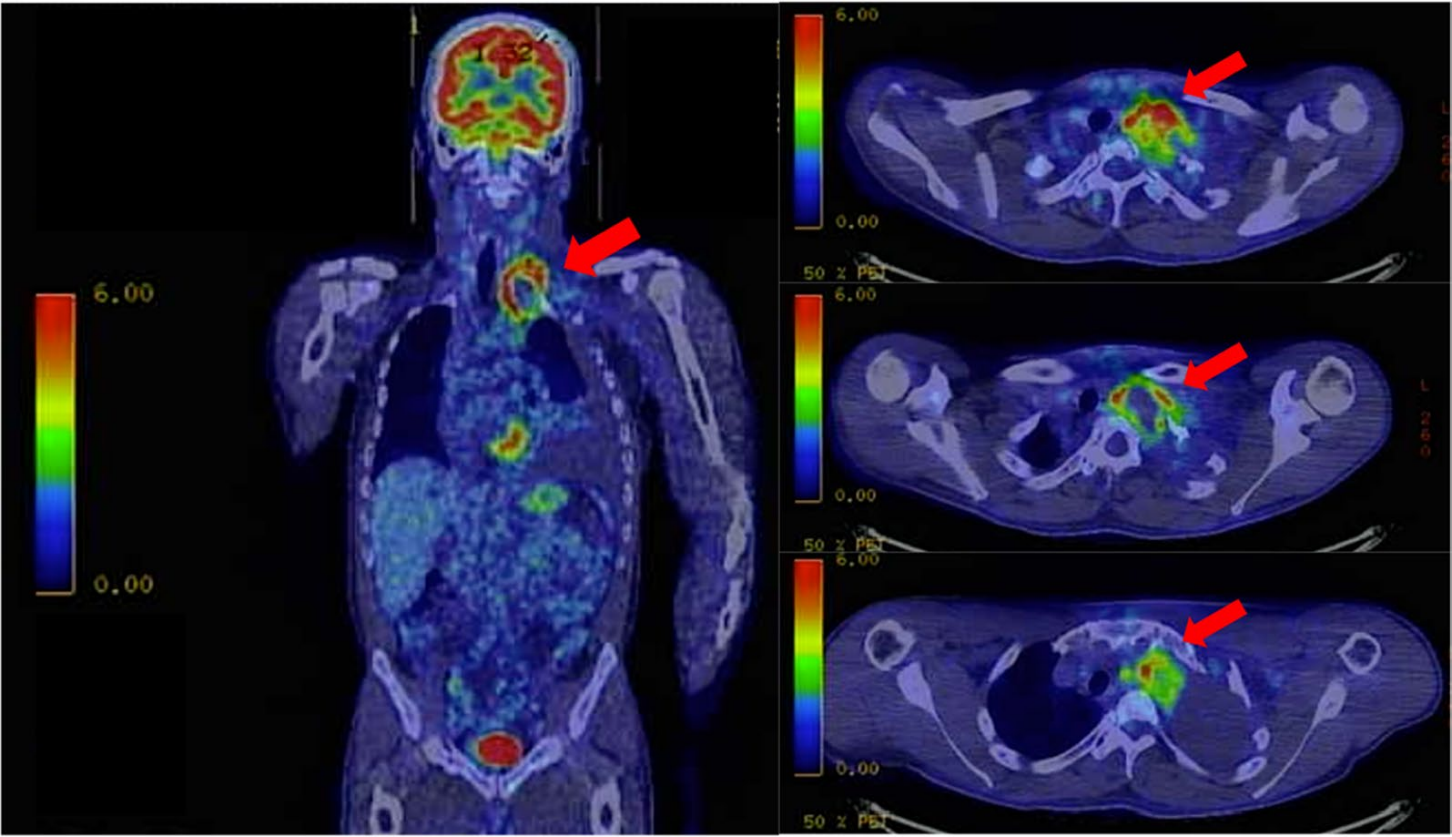
Fluorodeoxyglucose positron emission tomography–computed tomography (FDG-PET–CT) scan. FDG-PET–CT scan identifies a 38 × 53 mm mass in the left upper mediastinum, primarily with limbal accumulation

A 32-year-old man developed persistent left back and upper arm pain and presented to the clinic 3 days after onset. Chest radiography showed left pleural effusion and an opacity at the apex of the left lung (Fig. 1), prompting referral to the pulmonology department the next day. He exhibited scattered café-au-lait spots and left-sided Horner syndrome characterized by ptosis and miosis. Computed tomography revealed a mass in the left upper mediastinum. Pleural puncture yielded bloody fluid (hemoglobin, 9.0 mg/dL), and the pleural effusion was sent for cell block analysis. Subsequent chest radiography revealed no increase in the left pleural effusion. With no evidence of acute bleeding, fluorodeoxyglucose positron emission tomography–computed tomography (FDG-PET–CT) was scheduled to evaluate the mass the next day. The FDG-PET–CT scan identified a 38 × 53 mm mass in the left upper mediastinum, with an SUV max of 7.7 primarily accumulated at the margins (Fig. 2). These findings suggested an upper mediastinal tumor without lymph node involvement or distant metastasis. CE-CT was performed the next day for close examination, which revealed an approximately 25 mm contrast-enhancing mass in the left upper mediastinum, indicating a pseudoaneurysm of the left subclavian artery. The patient was transferred the same day for treatment of the subclavian pseudoaneurysm.

**Fig. 3:**
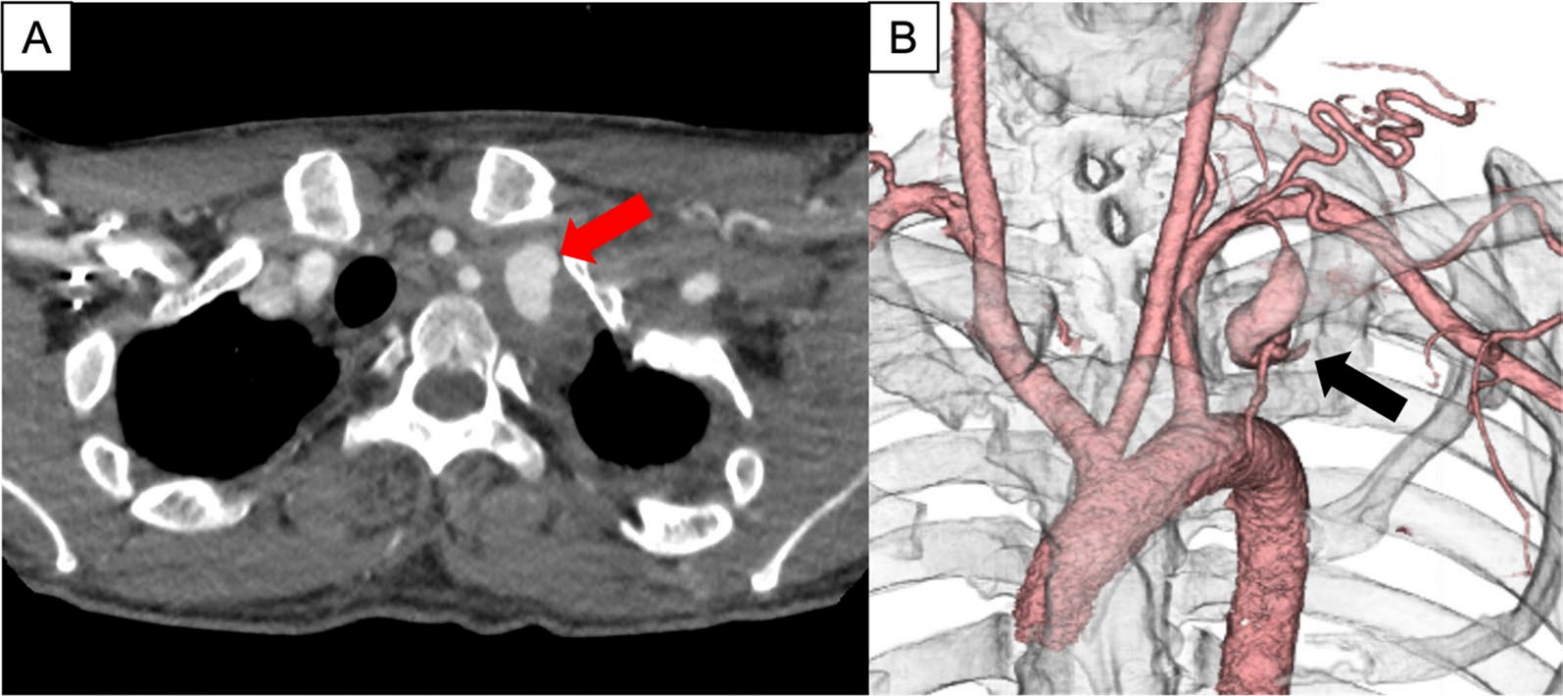
Computed tomography scan. In the axial image from the computed tomography scan, the red arrow indicates the left internal thoracic artery aneurysm (**A**). In the three-dimensional computed tomography reconstruction, the black arrow indicates the left internal thoracic artery aneurysm (**B**)

Upon arrival at our hospital, vital signs were stable, and chest radiography revealed no pleural effusion. Blood tests showed a hemoglobin of 9.7 mg/dL and hematocrit of 28.6%. The patient met three of the National Institutes of Health diagnostic criteria for NF-1, including café-au-lait spots, spinal curvature, and family history. A 1-mm slice CE-CT scan was performed for surgical planning, and the presence of a hematoma around the aneurysm and the clinical course led to a diagnosis of a left ITAA with contained rupture (Fig. 3).

**Fig. 4:**
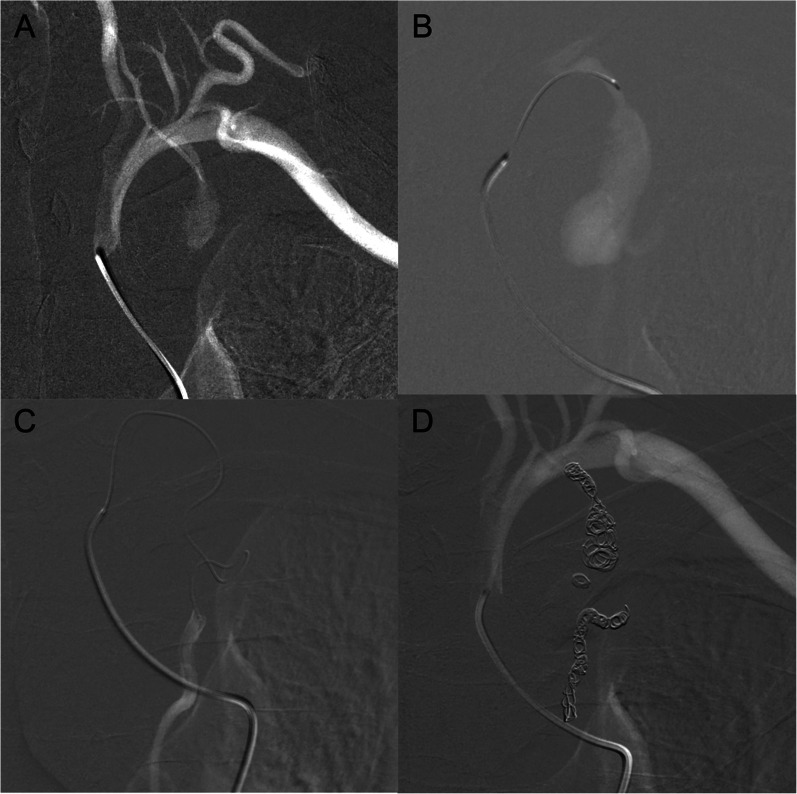
Angiography. The subclavian artery is cannulated using a 4 Fr hook-shaped catheter. Contrast injection reveals a contained rupture of the left internal thoracic artery aneurysm with no extravasation (**A**). A 1.9 Fr catheter is inserted into the left internal thoracic artery and contrast is administered (**B**). The distal portion of the aneurysm is successfully cannulated using a 1.9 Fr catheter (**C**). The final angiogram confirms successful embolization of the internal thoracic artery at both the proximal and distal ends using several microcoils, each measuring 3–4 mm in diameter (**D**)

On the fourth day after the initial outpatient visit, given the risk of re-rupture, emergency angiography was performed via the right femoral artery. The subclavian artery was cannulated using a 4 Fr hook-shaped catheter (C2; Medikit, Tokyo, Japan) and contrast angiography revealed a left ITAA without extravasation (Fig. 4A). Thereafter, through the 4 Fr catheter, a 1.9 Fr catheter (Carnelian MARVEL Non-Taper 1.9/1.9 Fr; Tokai Medical Products, Inc., Aichi, Japan) was inserted into the left internal thoracic artery (ITA) using a 0.014-in. guidewire (CHIKAI V 014; Asahi Intecc, Aichi, Japan) (Fig. 4B). This guidewire was passed through the distal portion of the aneurysm, allowing for successful cannulation with a 1.9 Fr catheter (Fig. 4C). The left ITA was successfully embolized at both the proximal and distal ends using several microcoils (Target 360 Ultra/Target Helical Ultra; Stryker Corp., Michigan, USA), each measuring 3–4 mm in diameter. The final angiogram confirmed successful ITA embolization (Fig. 4D).

The patient was admitted to the intensive care unit and transferred to the ward the following day. Postoperative CE-CT revealed a thrombosed ITAA with no extravasation, and the patient was discharged on the 4th postoperative day. At the 6-month follow-up, the ITAA had shrunk, and the patient was in good general condition.

## Discussion

We successfully treated a patient with NF-1 who presented with a ruptured left ITAA. The ITAA rupture manifested as a hemothorax, followed by spontaneous hemostasis. Unlike previous reports that consistently showed an acute progression [4–10], this case presented a subacute clinical course. The diagnosis was challenging and time-consuming, but was ultimately reached. The small size and tumor-like appearance of an ITAA make diagnosis difficult [11]. The PET–CT findings also complicated the diagnosis, but the accumulation was due to inflammation around the ruptured aneurysm. Therefore, vascular abnormalities should also be considered when identifying mediastinal tumors. In this case, CE-CT was useful in diagnosing an aneurysm, and thin-slice CE-CT was especially crucial for differentiating a subclavian artery aneurysm from an ITAA.

Only seven cases of ruptured ITAA associated with NF-1 have been reported [4–10]. Two patients were hemodynamically unstable and underwent surgery [4, 5], another two received a combination of endovascular treatment and surgery [6, 7], while the remaining three were managed exclusively with endovascular treatment [8–10]. Only the latter five patients survived. To date, there are no reports indicating whether surgery or endovascular treatment is superior for patients with stable vitals, but there are many reports of successful endovascular treatments [8–10]. Therefore, we opted for endovascular treatment with surgical backup. If peripheral coil embolization of the ITAA was not feasible or if a residual leak remained, we planned to perform ITA clipping via left anterior thoracotomy. Fortunately, the aneurysm treatment was successfully completed using coil embolization. Data on the long-term prognosis of coil embolization for ITAA in patients with NF-1 are scarce and require careful outpatient follow-up.

## Conclusions

This case highlights the importance of considering vascular lesions in NF-1 patients who present with pleural effusion. It also emphasizes the challenges in diagnosing ITAA and the effectiveness of thin-slice contrast-enhanced computed tomography and endovascular treatments.

## Data Availability

Not applicable.

## Abbreviations

CE-CT: Contrast-enhanced computed tomography
FDG-PET–CT: Fluorodeoxyglucose positron emission tomography–computed tomography
ITA: Internal thoracic artery
ITAA: Internal thoracic artery aneurysm
NF-1: Neurofibromatosis type 1
